# Research trends and hotspots of neuropathic pain in neurodegenerative diseases: a bibliometric analysis

**DOI:** 10.3389/fimmu.2023.1182411

**Published:** 2023-07-12

**Authors:** Yujie Fu, Chan Gong, Chenchen Zhu, Weiquan Zhong, Jiabao Guo, Binglin Chen

**Affiliations:** The Second Clinical Medical College, Xuzhou Medical University, Xuzhou, China

**Keywords:** bibliometric, neuroinflammation, microglia, neuropathic pain (NP), neurodegenerative diseases

## Abstract

**Background:**

Neuropathic pain is caused by a neurological injury or disease and can have a significant impact on people’s daily lives. Studies have shown that neuropathic pain is commonly associated with neurodegenerative diseases. In recent years, there has been a lot of literature on the relationship between neuropathic pain and neurodegenerative diseases. However, bibliometrics is rarely used in analyzing the general aspects of studies on neuropathic pain in neurodegenerative diseases.

**Methods:**

The bibliometric analysis software CiteSpace and VOSviewer were used to analyze the knowledge graph of 387 studies in the Science Citation Index Expanded of the Web of Science Core Collection Database.

**Results:**

We obtained 2,036 documents through the search, leaving 387 documents after culling. 387 documents were used for the data analysis. The data analysis showed that 330 papers related to neuropathic pain in neurodegenerative diseases were published from 2007–2022, accounting for 85.27% of all published literature. In terms of contributions to the scientific study of neuropathic pain, the United States is in the top tier, with the highest number of publications, citations, and H-indexes.

**Conclusion:**

The findings in our study may provide researchers with useful information about research trends, frontiers, and cooperative institutions. Multiple sclerosis, Parkinson’s disease, and Alzheimer’s disease are the three most studied neurodegenerative diseases. Among the pathological basis of neurodegenerative diseases, microglia-regulated neuroinflammation is a hot research topic. Deep brain stimulation and gamma knife radiosurgery are two popular treatments.

## 1 Introduction

Neurodegenerative diseases are characterized by neurodegenerative changes or apoptosis which can worsen over time and lead to functional impairment (1). Common neurodegenerative diseases include multiple sclerosis (MS), Parkinson’s disease (PD), Alzheimer’s disease (AD), Huntington’s disease and Amyotrophic lateral sclerosis. Studies have shown that patients with neurodegenerative diseases often present with pain and that the pain described by some patients with neurodegenerative diseases may be related to neuropathic pain (NP) (2).

NP is a chronic condition caused by dysfunction of the central or peripheral nervous system and its symptoms include spontaneous pain, nociceptive hyperalgesia and tactile allodynia (3). As a common form of pain in neurodegenerative diseases, NP causes chronic physical and psychological suffering and places a tremendous burden on patients, physicians and the health care system (4). However, due to the specific nature of neurodegenerative diseases (e.g. cognitive dysfunction), patients are less able to recognize and self-report pain (5), which increases negative emotions in patients with neurodegenerative diseases and not only further aggravates their cognitive dysfunction, but also seriously affects their quality of life and expected medical outcomes. It is therefore necessary to focus on pain in patients with neurodegenerative diseases. Studies have shown that abnormal activation of central microglia (6), neuroinflammation (7, 8), demyelinating lesions (9), altered cell signaling (10, 11), and acquired senescence/cell death (12) are mechanisms that contribute to the pathogenesis of neurodegenerative diseases and that some of these mechanisms are also mechanisms that induce NP, suggesting a close link between the two (13).

Bibliometric analysis is an emerging tool to obtain more quantitative information on cooperation between various organizations, the influence of publications, and developing trends (14, 15). At present, there are no bibliometric analysis papers focusing on the role of NP in neurodegenerative diseases. Therefore, we conducted a detailed search of the Web of Science Core Collection Database and analyzed the results through Microsoft Excel (version 2019), CiteSpace (version 6.1.R6 and version 5.6.R5) and other software to summarize previous research results and provide references for subsequent studies.

## 2 Materials and methods

### 2.1 Search strategy

The data of this study came from the Science Citation Index-Expanded of the Web of Science Core Collection Database. We conducted a literature search on January 2, 2023, and 2,036 pieces of literature were retrieved. The retrieval strategy was as follows: TS= (“Neuropathic Pain” OR “Neuralgia”) AND TS = (“multiple sclerosis” OR “amyotrophic lateral sclerosis” OR “Parkinson’s” OR “Parkinson disease” OR “Alzheimer’s” OR “Alzheimer disease” OR “Huntington’s” OR “Huntington disease” OR Neurodegenerative). The papers obtained were from 1994–2022 and the type of literature was an article or a review. The search strategy identified papers that mention these terms in the title, abstract, author keywords, or keywords plus. After that, we checked the titles, abstracts, author keywords, and keywords plus of all the documents, and eliminated those that were not relevant. Finally, 387 documents remained after elimination, and all of them were identified by CiteSpace.

### 2.2 Analytical tool

We extracted the literature downloaded from the Science Citation Index-Expanded of the Web of Science Core Collection Database using Excel, and the extracted data included full records and the references cited. CiteSpace (16) is a visual analysis software developed by Dr. Chao-Mei Chen based on the theory of citation analysis. We used the software to transform quantitative literature data into visual maps and networks that provide key information including research hotspots, frontiers, and country cooperation between countries/regions/institutions. There are different nodes and links in various CiteSpace visual graphs. The nodes can represent different keywords, countries, institutions, journals, etc. The larger the node, the more emergence or citations in this area. The links between nodes represent their relationships. When countries/regions/institutions represented by node appear in the same document, there is a connecting line between the two. Different colors represent different years of issue. The newer the date, the warmer the color of the node/link, and the earlier the date, the cooler the color of the node/link. The centrality indicates the importance of this node in the network, and in CiteSpace, nodes with purple rings are considered to be centrally located with high centrality. In addition, we used VOSviewer (version 1.6.18) to assist in analysis and mapping.

## 3 Results

### 3.1 Analysis of publication outputs

**Figure 1:**
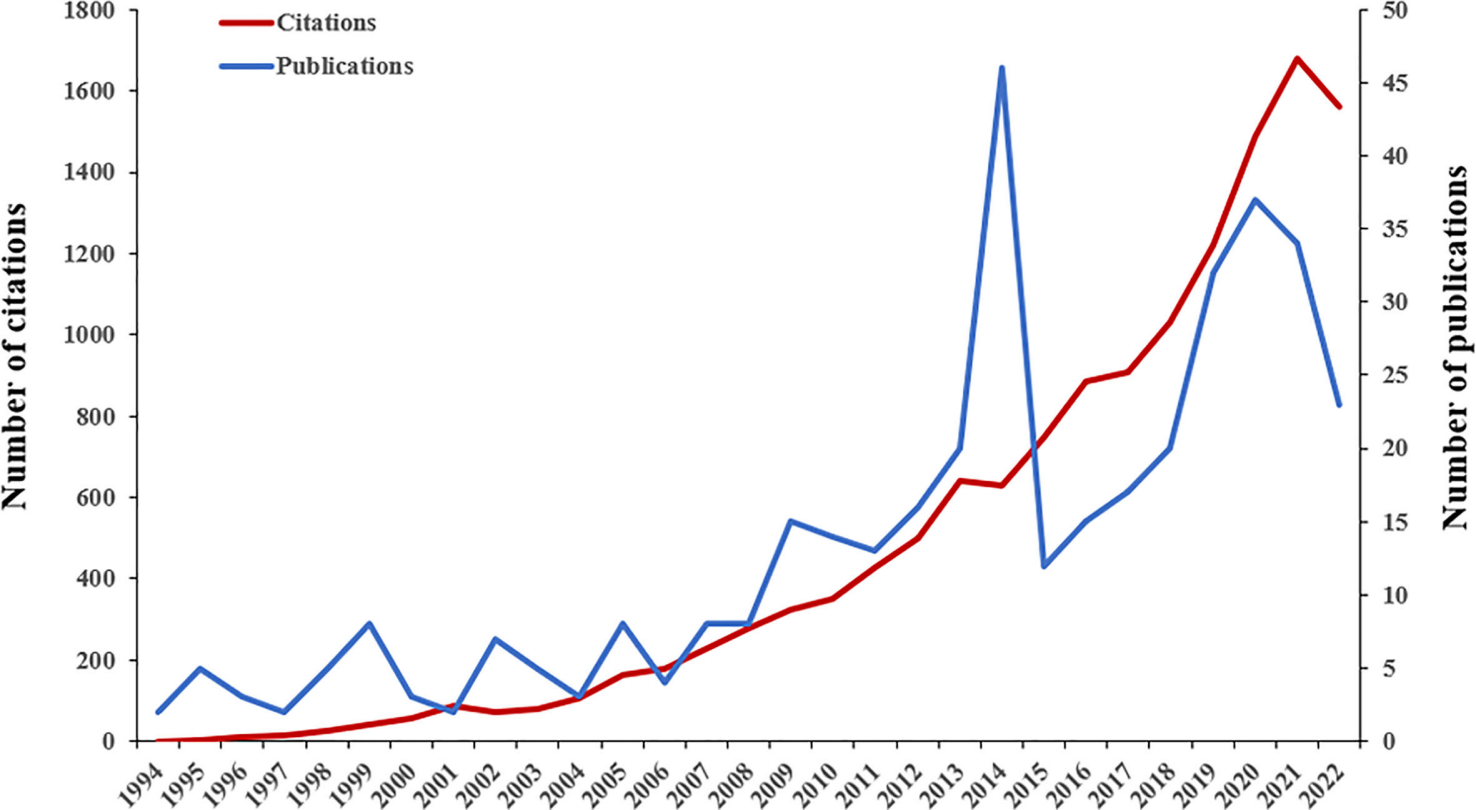
The number of publications and citations per year.

A total of 387 papers from 1994 to 2022 were eventually used for data analysis. According to the time trend analysis (**Figure 1**), the publications increased from 2 in 1994 to 23 in 2022. The number of publications fluctuated. The growth of publications in the literature could be roughly divided into three stages (1994–2006, 2007–2014, and 2015–2022). The number of publications fluctuated at a lower level from 1994 to 2006. Next, the number of publications increased fluctuating from 2007 to 2014 and peaked in 2014. In the third stage, there was a largely year-on-year trend of increasing numbers in 2015–2020, but in 2022 there was a large drop in the number of publications. Although the second and third stages covered only 8 years, the number of articles published reached 140 and 190 respectively, far exceeding the first stage. In total, the papers published in the third stage accounted for 49.10% of all published literature. **Figure 1** also shows a gradual increase in the number of citations to the literature. 387 papers were cited 13,731 times and the average number of citations per item was 35.48.

### 3.2 Analysis of countries and institution

**Figure 2:**
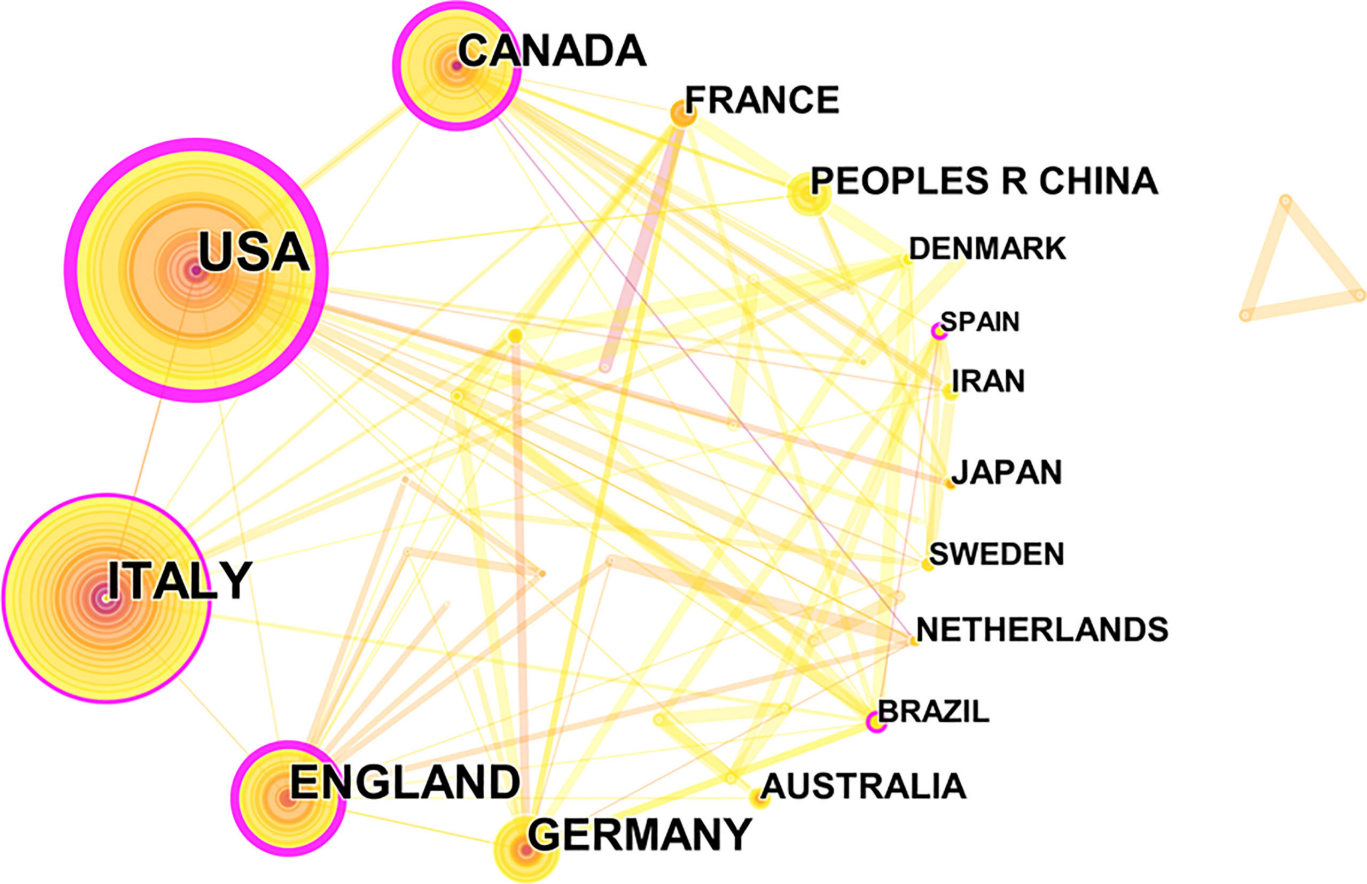
Map of co-countriesco-regions.

The 387 papers were contributed by 51 different countries/regions. The top ten countries/regions by number of publications are shown in **Table 1**. According to the number of publications, the United States (83) was the most prolific country, followed by Italy (69), England (43), and Canada (41). The United States also had the highest number of citations (3,113) and H-index (32). In order of centrality, the top three countries were the United States (0.42), Italy (0.18), and England (0.28). However, some prolific countries such as Germany, France, and China had a centrality of less than 0.1. **Figure 2** shows the cooperation links between each country/region.

**Table 1:** Top ten most productive countries/regions.

Countries/Regions	Publications	Citations	Average citations per item	Centrality	H-index
United States	83	3,113	37.51	0.42	32
Italy	69	2,699	39.12	0.18	28
England	43	2,160	50.23	0.28	23
Canada	41	1,459	35.59	0.23	19
Germany	38	1,002	26.37	0.08	16
France	19	654	34.42	0.04	13
China	19	184	9.68	0.01	8
Australia	15	652	43.47	0.04	11
Japan	14	258	18.43	0.00	9
Brazil	13	443	34.08	0.11	8

**Figure 3:**
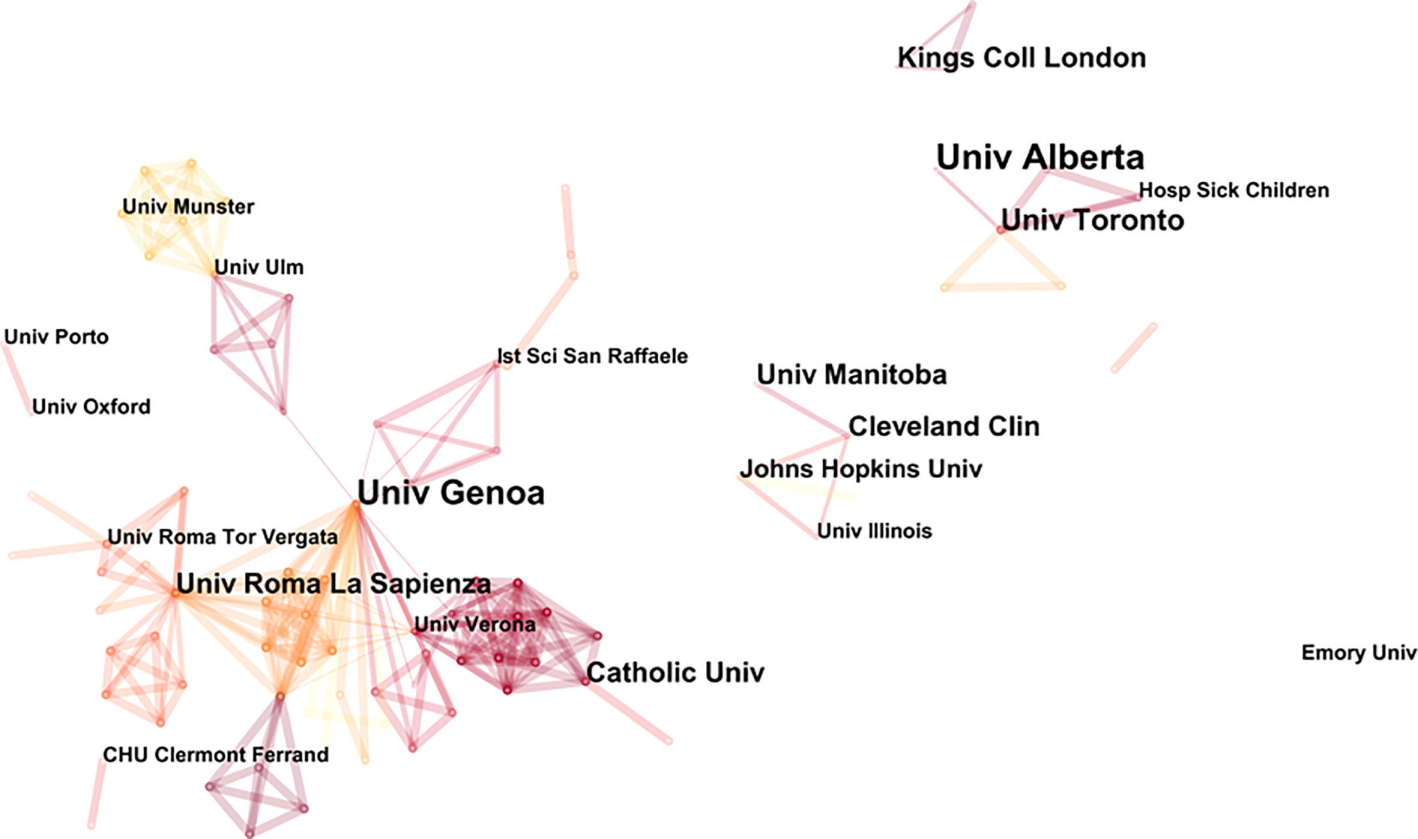
Map of co-institutions.

Prolific institutions in publications on NP in neurodegenerative diseases are presented in **Table 2**. The most prolific institution was the University of Alberta (9 publications), followed by the University of Genoa (8 publications) and the University of Toronto (6 publications). Most of the top ten most prolific institutions were from Canada and the United States. **Figure 3** illustrates the partnership between the different institutions. The University of Genoa and the Sapienza University of Rome were two important nodes in this network.

**Table 2:** Top ten most productive institutions.

Rank	Institution	Country	Publications	Centrality
1	University of Alberta	Canada	9	0.00
2	University of Genoa	Canada	8	0.03
3	University of Toronto	Canada	6	0.00
4	Aarhus University Hospital	Denmark	6	0.00
5	Sapienza University of Rome	Rome	5	0.02
6	Cleveland Clinic Foundation	United States	5	0.00
7	Iran University of Medical Sciences	Iran	5	0.00
8	King’s College London	England	5	0.00
9	Karolinska Institutet	Sweden	5	0.00
10	Catholic University of the Secret Heart	United States	5	0.00

### 3.3 Analysis of journals

The 387 papers were published in 197 different journals. The top ten productive journals in publishing articles or reviews on NP in neurodegenerative diseases are shown in **Table 3**. They accounted for 27.13% (105 publications) of the total publication outputs. Through the results of journals, we found that Pain published the highest number of literatures (19, 4.910%), followed by European Journal of Pain (13, 3.359%), Journal of Neurosurgery (12, 3.101%), and Neurology (12, 3.101%). Among the top 10 journals, Pain was cited the most and Neurology was cited the second most, with these two journals being cited far more often than others. Neurology also had the highest average citations per item (111.17; IF2021, 11.8).

**Table 3:** Top ten most productive journals.

Journals	Publications	Citations	Average citations per item	2021 IF
Pain	19	1,345	70.79	7.926
European Journal of Pain	13	586	45.08	3.651
Journal of Neurosurgery	12	284	23.67	5.408
Neurology	12	1,334	111.17	11.800
Neurological Sciences	11	176	16.00	3.830
Neurosurgery	9	235	26.11	5.315
Multiple Sclerosis and Related Disorders	8	55	6.88	4.808
World Neurosurgery	8	119	14.88	2.210
Pain Medicine	7	158	22.57	3.637
Stereotactic and Functional Neurosurgery	6	215	35.83	1.643

### 3.4 Analysis of authors

The 387 papers on NP in neurodegenerative diseases were contributed by 1,871 authors. **Table 4** shows the top ten authors by publication. Among the most prolific authors, Solaro, Claudio ranked the first with 12 publications, followed by Kerr, Bradley J. (8 publications), Truini, Andrea (8 publications) and Cruccu, Giorgio (7 publications). Solaro, Claudio had the most citations (403), followed by Franzini, Andrea (351). Franzini, Andrea had the highest number of average citations per item, far more than anyone else.

**Table 4:** Top ten most productive authors.

Author	Publications	Citations	Average citations per item	H-index
Solaro, Claudio	12	403	33.58	8
Kerr, Bradley J.	8	273	34.13	7
Truini, Andrea	8	341	42.63	7
Cruccu, Giorgio	7	324	46.29	7
Tenorio, Gustavo	7	151	21.57	7
Moalem-Taylor, Gila	6	106	17.67	5
Centonze, Diego	5	270	54.00	5
De Andrade, D. Ciampi	5	158	31.60	4
Franzini, Andrea	5	351	70.20	4
Perera, Chamini J.	5	102	20.40	5

### 3.5 Analysis of the 10 most-cited papers

The number of citations of a study’s published literatures can be used to demonstrate its academic impact on the field of study. **Table 5** lists the ten most-cited papers and the average top ten cited papers per year on NP in neurodegenerative diseases. The ten most-cited papers ranged from 205 to 447, and the combined citations of the ten most-cited papers accounting for 22.23% of the total citations of 387 publications. The most-cited paper was “Randomized, controlled trial of cannabis-based medicine in central pain in multiple sclerosis” by Rog, David J, published in Neurology in 2005.

**Table 5:** The top 10 papers based on the number of citations.

Title	Journal	Year	Citations
Total			
Randomized, controlled trial of cannabis-based medicine in central pain in multiple sclerosis	Neurology	2005	447
Tumor Necrosis Factor Alpha: A Link between Neuroinflammation and Excitotoxicity	Mediators of Inflammation	2014	392
Cannabinoids and the expanded endocannabinoid system in neurological disorders	Nature reviews neurology	2020	324
Gabapentin: pharmacology and its use in pain management	Anaesthesia	2002	320
Pain in Parkinson’s disease: Prevalence and characteristics	Pain	2009	319
Does the cannabinoid dronabinol reduce central pain in multiple sclerosis? Randomised double blind placebo controlled crossover trial	BMJ-British Medical Journal	2004	308
Pain associated with multiple sclerosis: Systematic review and proposed classification	Pain	2008	286
Pain in Parkinson’s Disease	Movement Disorders	2010	235
Central pain in multiple sclerosis - prevalence and clinical characteristics	European Journal of Pain	2005	217
Chronic neuropathic pain is accompanied by global changes in gene expression and shares pathobiology with neurodegenerative diseases	Neuroscience	2002	205
Average per year			
Cannabinoids and the expanded endocannabinoid system in neurological disorders	Nature Reviews Neurology	2020	81.00
Tumor Necrosis Factor Alpha: A Link between Neuroinflammation and Excitotoxicity	Mediators of Inflammation	2014	39.20
Randomized, controlled trial of cannabis-based medicine in central pain in multiple sclerosis	Neurology	2005	23.53
Pain in Parkinson’s disease: Prevalence and characteristics	Pain	2009	21.27
Pain associated with multiple sclerosis: Systematic review and proposed classification	Pain	2008	17.88
Pain management in patients with dementia	Clinical Interventions in Aging	2013	17.64
Mast cells, glia and neuroinflammation: partners in crime?	Immunology	2014	16.90
Pain in Parkinson’s Disease	Movement Disorders	2010	16.79
Does the cannabinoid dronabinol reduce central pain in multiple sclerosis? Randomised double blind placebo controlled crossover trial	BMJ-British Medical Journal	2004	15.40
Gabapentin: pharmacology and its use in pain management	Anaesthesia	2002	14.55

Among the 10 most-cited papers, only one was from the last five years. “Cannabinoids and the expanded endocannabinoid system in neurological disorders” by Cristino, Luigia, published in Nature reviews neurology is the most recent and highly cited literature in the field, as well as the most cited on average per year. These highly cited papers focus mainly on the prevalence of neurodegenerative diseases, the characteristics of the disease such as NP, and the treatment of NP.

### 3.6 Analysis of keywords

**Figure 4:**
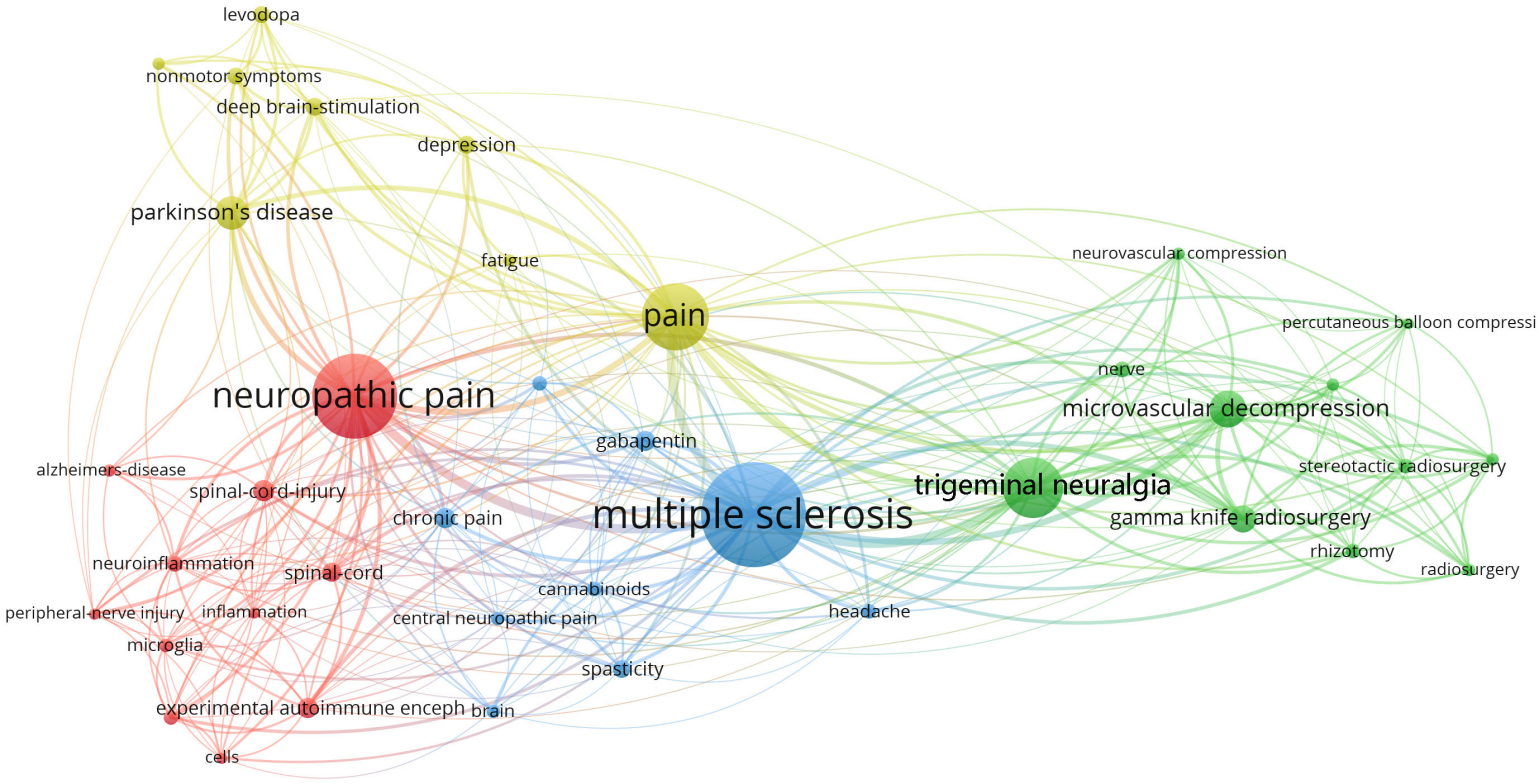
Map of Keyword co-occurrence.

**Figure 5:**
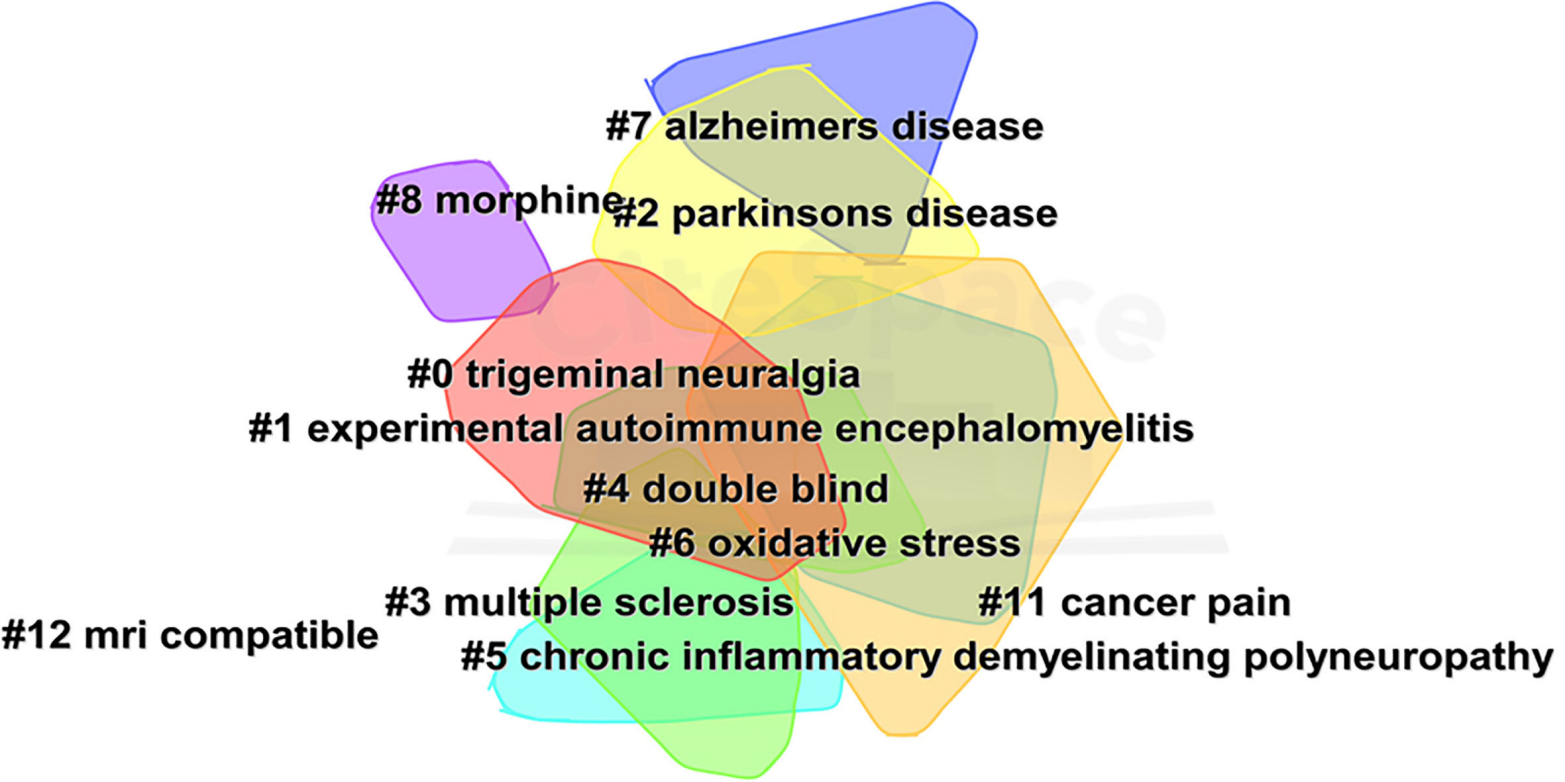
The cluster view of keywords.

**Figure 6:**
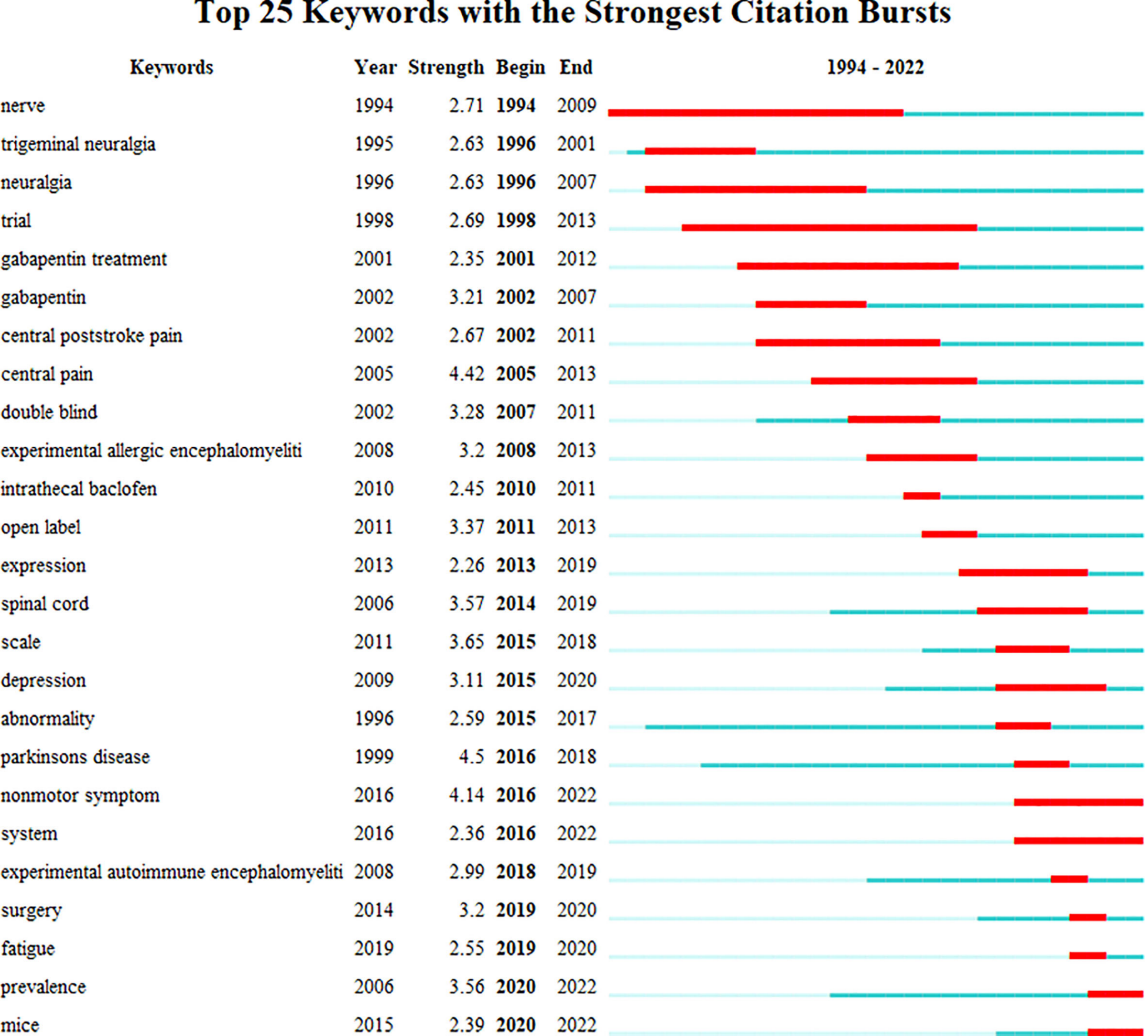
The keywords with the strongest citation bursts of publications on NP in neurodegenerative disease research.

**Figure 4** shows the network of keyword co-occurrence. Nodes represent different keywords, and lines represent relationships between keywords. Red, yellow, blue, and green represent the four categories centered on “neuropathic pain,” “pain,” “multiple sclerosis,” and “trigeminal neuralgia,” respectively. “Multiple sclerosis,” “neuropathic pain,” “pain,” and “trigeminal neuralgia” were the four most important keywords in this network. Current research keywords could be classified into eleven clusters, as shown in **Figure 5** (Unreasonable clusters such as #9 and #10 have been eliminated). The larger the number of cluster labels, the more documents are included under that cluster. **Figure 6** shows the top 25 keywords with the strongest citation bursts since 1994. Keywords with citation bursts can reflect the development of a knowledge field. Burst terms can reflect the research frontiers in the field. From **Figure 6**, the time of the beginning and end of the research frontier can be seen, and the length of the red bar shows the lasting time of the research frontier. The strength of the outbreak represents the intensity of the mutation. “Parkinson’s disease,” “central pain,” and “nonmotor symptom” were the top three keywords with the strongest citation. The newest research frontiers were “nonmotor symptom,” “system,” “prevalence,” and “mice”.

## 4 Discussion

### 4.1 Global research trends on NP

This study presents a bibliometric analysis of NP in neurodegenerative diseases between 1994 and 2022. The number of publications has shown a slow but erratic growth trend over time. It grew rapidly from 2013 to 2014, peaking in 2014. 2015 saw an abrupt decrease, and the number of publications per year increased from 2016 to 2020 and then decreased in 2021. The citation frequency of the literature has been increased year by year, showing an upward trend, with the largest increase in 2017–2021. The top three countries/regions in terms of the number of publications and citations were the United States, Italy, and England. The three countries/regions with the highest number of average citations per item were England, Australia, and Italy. The United States had the largest number of publications, citations, centrality, and H-index, indicating its significant academic influence in the field. The fact that nearly hundreds of countries around the world contribute to the field indicates that research in this area has a broad global reach. The University of Genoa and Sapienza University of Rome had academic collaborations with many other institutions and were two important nodes in the network of institutional co-occurrence (**Figure 3**). Except the University of Genoa and the Sapienza University of Rome, the other institutions had less centrality and future research needs to strengthen collaboration between core institutions in different countries. Among the top ten active journals, Pain was the most cited (1,345 citations) and Neurology had the highest number of average citations per item (111.17 citations; IF2021, 11.8), Pain also had the most publications (19).

### 4.2 Research hotspots and frontiers on NP

#### 4.2.1 Relationship between MS, PD, AD and NP

Among the top ten papers cited (**Table 5**), the paper titled, “Chronic neuropathic pain is accompanied by global changes in gene expression and shares pathobiology with neurodegenerative diseases”, was published by Wang, H et al. in Neuroscience in 2002. This study proposed that NP and neurodegenerative diseases shared common pathophysiological characteristics. In recent years, studies have shown that microglia-mediated neuroinflammation, which is an important pathological basis for diseases including neurodegenerative diseases and NP, is a hot topic of interest for researchers. Interestingly, microglia and neuroinflammation were also found to connect NP to MS, PD, and AD in our keyword co-occurrence map (**Figure 4**), suggesting a potential link between neurodegenerative diseases and NP (**Table 6**).

**Table 6:** The common pathophysiological characteristics between neuropathic pain and Multiple sclerosis, Alzheimer’s disease, Parkinson’s disease and Huntington’s disease.

Multiple sclerosis	Parkinson’s disease	Alzheimer’s disease	Huntington’s disease
•Neuroinflammation •Neurodegeneration (axon damage, neuron loss) •Synaptic and neural network defects •Spinal cord abnormality	•Neuroinflammation •Neurotransmitter concentration changes •Neuronal apoptosis	•Neuroinflammation •Neurotransmitter concentration changes •Neuronal apoptosis	•Neuroinflammation •DNA and RNA defects •Neurotransmitter concentration changes •Mitochondrial damage •Neuronal apoptosis

**Figure 7:**
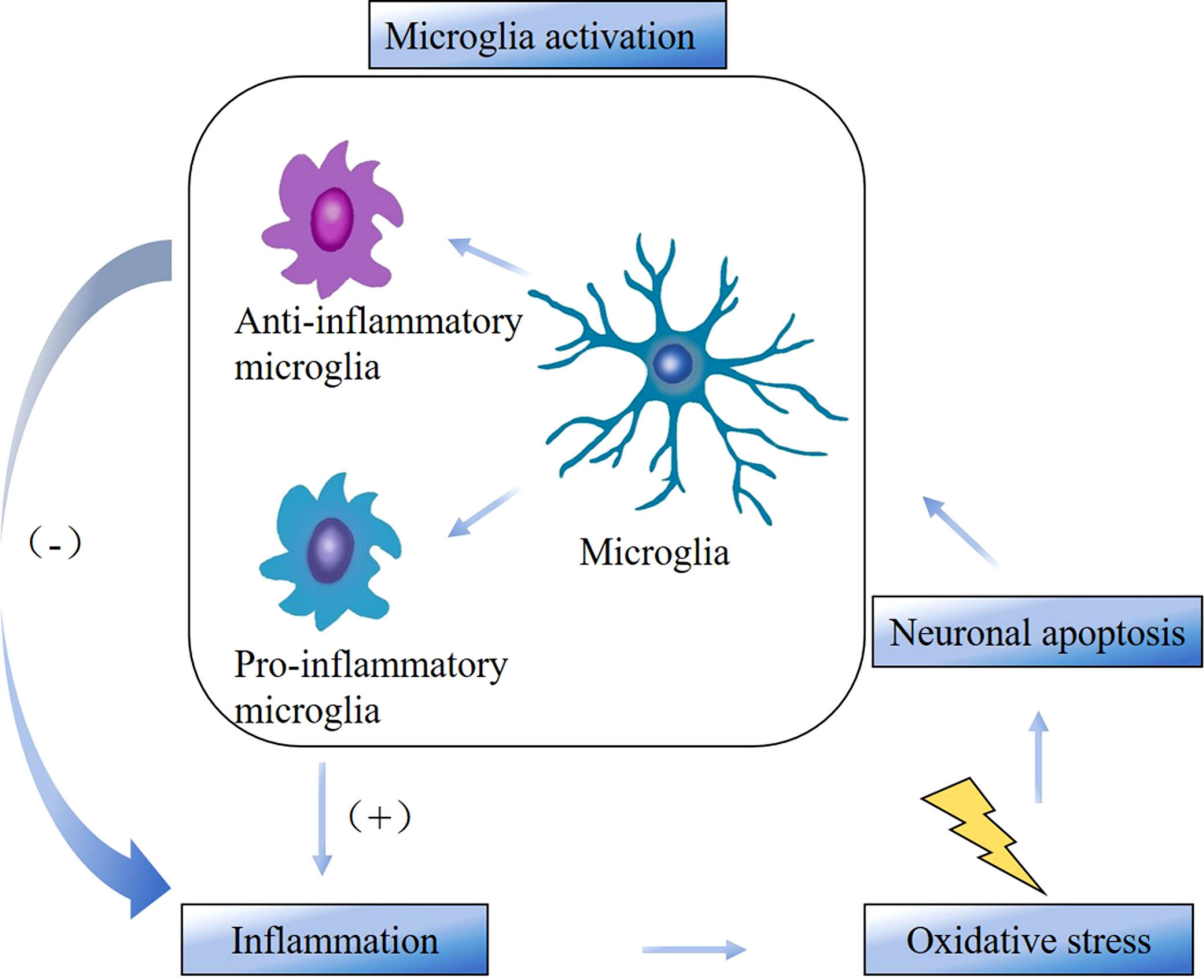
The cycle between microglia activation, inflammation, oxidative stress, and neuronal apoptosis.

Microglia-mediated neuroinflammation is an important pathological basis for the development of neurodegenerative diseases and is also closely associated with disease progression. Neuroinflammation is an immune response activated by microglia and astrocytes in the central nervous system (brain and spinal cord) and usually associated with direct damage to the nervous system. Neuroinflammation is present in MS, PD, AD and many other neurodegenerative diseases (17). Microglia are a major component of the intrinsic immune system of the brain and spinal cord and can respond rapidly when the body is exposed to injury or infection. Studies have shown that microglia could regulate neuroinflammation associated with neurodegenerative pathologies (18). Neurodegenerative diseases may involve neuronal apoptosis, which stimulates microglia activation and promotes inflammation. Persistent inflammation generates excess free radicals, which further exacerbate oxidative stress thereby generating excess reactive oxygen species and causing irreversible cellular damage. Cellular damage leads to cytokine necrosis, which causes apoptosis and further aggravates neuroinflammation. This forms a recurrent cycle (**Figure 7**) that eventually induces central and peripheral sensitization, leading to NP (19, 20). According to the keyword analysis, MS, PD, and AD were the three categories of neurodegenerative diseases with the highest attention in this field.

MS is a chronic immune-mediated inflammatory demyelinating disease of the central nervous system and is one of the common neurodegenerative diseases (21). The main symptom of the disease is loss of motor and sensory function, and it is now becoming recognized that chronic pain is also a major problem that affects 50% to 80% of patients with MS. A highly cited systematic review by O’Conner et al. found that the point prevalence of pain in MS patients approached 50%, with approximately 75% of patients reporting pain within one month of assessment (22). Pain is a common complication of MS (23), and NP is a major mechanism of increased risk of pain in patients with MS (24). A systematic review showed an overall prevalence of chronic pain of 52.1% in 274 patients, with the lower extremities being most frequently involved (36.9%). Of these patients, 89 had NP, with an overall prevalence of 23.7%, and was associated with involvement of the sensory function system. Pain intensity was significantly higher in patients with NP than in patients without NP (25). Pain adversely affects family life, ability to work, etc., and pain in MS patients is affected by age, duration of the disease, degree of functional impairment, spasticity, depression, and fatigue (26, 27). Pain in MS patients includes extremity pain, trigeminal neuralgia (TN), Lhermitte’s sign, painful tonic spasms, back pain and headache (26, 28). Pain in MS is central in more than half of patients and is associated with mechanical or thermal nociceptive sensitization (29). In the co-occurrence map of keywords (**Figure 4**), TN was one of the most important nodes linked to MS. Meanwhile, TN was linked to “neurovascular compression” and “microvascular decompression”. TN is the most characteristic NP in MS. Possible causes of MS-related TN include the destruction of main afferent nerves by pontine plaques, neurovascular compression, and demyelinating lesions affecting the nerves. Other sensory disturbances, including persistent pain and sensory deficits, can be caused by damage to secondary neurons in the spinal trigeminal complex (30–32). **Figure 6** shows that “Experimental autoimmune encephalomyelitis” was a research frontier of interest for many researchers in 2018–2019. Because it shares many clinical and neuropathological characteristics with MS, it is widely used to study MS. Olechowski et al. found that inflammation and reactive glial cell proliferation could be key mediators of abnormal pain in female C57BL/6 mice immunized with myelin oligodendrocyte glycoprotein MOG (35–55), and that model could serve as a tool to study NP in MS (33, 34). Other clinical and preclinical model studies also confirmed that demyelination and inflammation played an important role in the pathogenesis of NP in MS, and the progression of MS was associated with abnormal activation of microglia and astrocytes (29).

PD is a multi-system, progressive, incurable neurodegenerative disorder (35), associated with the loss of dopaminergic neurons in the substantia nigra of the brain. As the disease progresses, motor impairment and nonmotor symptoms are a considerable burden of disease. The outbreak graph for keywords (**Figure 6**) shows that “nonmotor symptom” was one of the latest frontiers of research. Pain has been shown to begin with clinical episodes of PD or follow thereafter as a nonmotor feature of PD (36). Beiske et al. published the study titled, “Pain in Parkinson’s disease: Prevalence and characteristics” in Pain in 2009 (37). They conducted questionnaires, neurological examinations, and structured interviews with 176 family-living Parkinson’s patients, and found that pain was significantly more common in Parkinson’s patients compared to the general population. The prevalence of pain in the PD population is 40% to 85% (38). In patients with PD it is generally described as dystonic pain and non-dystonic pain. Of the non-dystonic sources of pain, there has been less research around central pain (39). Schestatsky et al. found that primary central pain in patients with PD was due to abnormal control of the effects of pain input on autonomic centers and that dysfunction may occur in dopamine-dependent centers that regulated autonomic function and inhibitory regulation of pain input (40, 41). Neuroinflammation is one of the hot spots of NP research in recent years and one of the important immune modulations in PD. It has been found that postmortem PD patients had a significant increase in reactive microglia in the substantia nigra (42) and significant activation of microglia had been observed in various animal models (43).This suggests that abnormal activation of microglia might lead to elevated pro-inflammatory factors and degeneration of dopaminergic neurons in the midbrain region, resulting in pain (44).

AD is also a common neurodegenerative disorder characterized by persistent neuroinflammation leading to overall cognitive decline and progressive loss of memory and reasoning skills (45). The amyloid cascade hypothesis proposes that the misfolded and aggregated β-amyloid protein (Aβ) triggers the pathological characteristics of AD, such as neuronal cell death (46, 47), which requires clearance to maintain normal neuronal function. Many patients with AD suffer from NP, and studies have shown that pain can cause cognitive dysfunction (48, 49), patients with pain in multiple parts of the body have a higher risk of dementia (50). Studies have shown that microglia-regulated neuroinflammation plays a key role in the pathogenesis of AD (51). Activated microglia have a dual role in the clearance of Aβ. Anti-inflammatory microglia activation contributes to Aβ clearance which reduces Aβ accumulation (17, 52), whereas pro-inflammatory microglia activation can induce dysregulated neuroinflammatory responses and leads to synapse loss, neuronal distress, and Aβ production (53, 54). The use of targeting microglia in the central nervous system can provide a new reference for diagnosis and intervention in AD.

#### 4.2.2 Treatment of NP

The treatment of NP in neurodegenerative diseases is a great challenge due to the heterogeneity of etiology, symptoms, and underlying mechanisms. Treatment of NP can be divided into pharmacological and non-pharmacological treatments. Non-pharmacological treatments can continue to be divided into invasive and non-invasive treatments.

The main drugs currently used to treat NP are nonsteroidal anti-inflammatory drugs, opioids, antidepressants, and anticonvulsants. Anticonvulsants and antidepressants are the recommended first-line treatment drugs. “Gabapentin” and “cannabinoids” were the two drugs that appear on the map of keyword co-occurrence (**Figure 4**), and “gabapentin” was also a frontier of research during 2001-2012. Among the top ten cited papers (**Table 5**), there were 4 papers related to “gabapentin” and “cannabinoids”. These four papers are also among the top ten averages citations per year.

Gabapentin is an anticonvulsant. Studies have shown that gabapentin was particularly effective in relieving allergic reactions and nociceptive sensitization in animal models (55). The drug had been clearly shown to be effective in the treatment of NP in diabetic neuropathy (56) and postherpetic neuralgia (57). Its more favorable side effects and lack of drug-drug interactions in different patients (including the elderly) made it an attractive drug (58). Therefore, gabapentin was once considered an important drug for the treatment of NP syndromes (58, 59). Available studies have shown that short-term use (three months) could lead to adverse cardiovascular disease (60) while long-term use of gabapentin could damage musculoskeletal conditions (61–63). Knowing the potential complications can have a considerable impact on the treatment plan given by the clinician. When gabapentin is limited by adverse effects, the cannabinoid is emerging as a promising drug for the treatment of NP. The analgesic effects of exogenous cannabinoids have been extensively demonstrated in experimental models of NP, and there is growing evidence that cannabinoids can provide analgesia in situations that are difficult to treat with other treatments (64–66). However, the duration of treatment and the route of administration that provides better tolerability and safety are still lacking in-depth studies (67). Most guidelines recommend the use of monotherapy as the first and second treatment options (68, 69). Many years ago, researchers have suggested that drug combination therapy is also justified because NP results from multiple mechanisms, but few studies of combination therapy have been documented (70). Combining different medications can sometimes improve analgesia and/or tolerability (71), but the increased use of combination medications also poses some safety concerns. In 2022, Hahn et al. (72) found that opioid-gabapentin combination therapy, despite reducing gastrointestinal adverse effects, may also lead to an increased risk of central nervous system depression and death. A recent meta-analysis (73) compared the efficacy of combinations of two or more drugs with placebo or at least one monotherapy in adults with NP. Their findings failed to demonstrate that the combination of opioid-antidepressants, opioid-gabapentin, and gabapentin-antidepressants was superior to the two monotherapies. Thus, in cases where monotherapy has limited efficacy, combination therapy may be used as a complementary treatment but also requires close monitoring of individual doses to ensure safety.

In the map of keyword co-occurrence (**Figure 4**), “microvascular decompression,” “gamma knife radiosurgery” were associated with MS and TN, and “deep brain stimulation” was associated with PD, suggesting that microvascular decompression, gamma knife radiosurgery, and deep brain stimulation were common non-pharmacological treatments for NP in neurodegenerative diseases such as MS and PD.

Among invasive treatments, microvascular decompression is considered a safe and effective vascular compression treatment for MS-related TN. It can also be recommended as the preferred procedure in some cases of MS (74). In addition, gamma knife radiosurgery is also an effective surgical treatment for MS-related TN (75–78).

In recent years, a number of non-invasive treatments have become popular with patients. Deep brain stimulation of the subthalamic nucleus has been shown to reduce the incidence of pain and improve the quality of life in patients with idiopathic PD with refractory motor fluctuations (79). In addition, studies have shown that anodal transcranial direct current stimulation reduced pain scores in patients with central chronic pain in MS, and this effect lasted beyond the stimulation period, resulting in long-term clinical effects (80, 81). Other studies have found that transcranial magnetic stimulation could improve motor and other symptoms associated with neurodegenerative processes such as NP, amyotrophic lateral sclerosis, MS, AD, PD, or Huntington’s disease (82).

Besides, exercise has been gaining popularity in recent years as a convenient and low-cost non-invasive treatment with multiple health benefits. In animal models of NP, several studies have found that exercise could reduce mechanical and thermal nociceptive sensitization (83, 84). Human studies have shown that moderate aerobic exercise, combining aerobic and resistance exercise, or high-intensity interval training, could reduce some NP. However, some indicators of NP such as thermal pain thresholds could not be improved (85). Exercise shows beneficial effects on NP in PD. Exercise acts on the muscle activity response, and myokines released during muscle contraction can modulate its activity by interacting with receptors in microglia. On the one hand, it can inhibit the pro-inflammatory mediators induced by microglia activation in PD, thus reducing the level of pro-inflammatory cytokines. On the other hand, it upregulates anti-inflammatory cytokines, which increases the anti-inflammatory level in the central nervous system and exerts neuroprotective activity (86). Exercise prevents cell death of dopaminergic neurons and modulates pain through dopaminergic and non-dopaminergic pain inhibitory pathways. However, it is not clear whether exercise exacerbates pain and impedes neuroplastic changes thereby affecting pain regulation in the central nervous system. In exercise training, there is still a need to avoid acute exacerbation of pain after exercise, while allowing for the establishment of physical activity tolerance (87). The pattern, intensity, frequency, and duration of exercise doses required for NP need to be systematically investigated (88).

The current treatment of NP in neurodegenerative diseases is mainly symptomatic, while mechanism-based treatments are lacking. Highly effective drugs are still lacking in pharmacological treatments.

## 5 Benefits and limitations

This study is the first bibliometric analysis of the literature related to NP in neurodegenerative diseases from 1994 to 2022. The data for the bibliometric analysis were obtained from the Science Citation Index-Expanded of the Web of Science Core Collection Database. 2,036 papers were retrieved from the database with a specific search strategy. By checking the titles, abstracts, author keywords, and keywords plus of all the documents, we eliminated the irrelevant papers and finally left 387 papers. A total of 197 different journals were included in the study, which is quite rich in data. In addition, the study covered a wide range of fields including neuroscience, surgery, pharmacology, immunology, and biochemistry. This study analyzed annual postings, prolific journals, articles with high citations, collaborations between different countries/institutions, research hotspots, and frontiers in the field. The findings in this study may provide researchers with useful information about research trends, frontiers, and cooperative institutions. Furthermore, CiteSpace and VOSviewer were used to transform quantitative literature data into visual maps and networks, making the information easy to understand. However, it was impossible to avoid the limitations of the study. Due to the format requirements of CiteSpace, all data in this study were only retrieved from the Science Citation Index-Expanded of the Web of Science Core Collection Database so the relevant publications may not be comprehensive. In addition, articles published after the search date could not be included in our analysis, leading to a lack of timeliness in the study.

## 6 Conclusion

This study demonstrates from a historical perspective that the field of research on NP in neurodegenerative diseases is well developed and has broad prospects, helping us realize the main research countries and institutions, core journals, overall development trend, hotspots, and research frontiers. Some prolific institutions with low centrality still need to strengthen further academic associations to expand their influence in this field. MS, PD, and AD are the three most studied neurodegenerative diseases. Among the pathological basis of neurodegenerative diseases, microglia-regulated neuroinflammation is a hot research topic. Due to the heterogeneity of NP in neurodegenerative diseases in terms of mechanism, etiology, symptoms, and treatment, its treatment remains challenging. Deep brain stimulation and gamma knife radiation therapy are two popular methods. Most of the current treatment revolves around the disease, and a mechanism-based approach to treatment is still lacking.

## Data availability statement

The original contributions presented in the study are included in the article/supplementary material. Further inquiries can be directed to the corresponding authors.

## Author contributions

YF was in charge of literature collation, analysis, and writing. CG and CZ was in charge of literature collation and article proofreading. WZ was in charge of proofreading. JG and BC was in charge of concept design, article proofreading and project guidance. All authors contributed to the article and approved the submitted version.
